# Vaginal Exposure to *Candida albicans* During Early Gestation Results in Adverse Pregnancy Outcomes *via* Inhibiting Placental Development

**DOI:** 10.3389/fmicb.2021.816161

**Published:** 2022-02-24

**Authors:** Zhiyong Dong, Chong Fan, Wenwen Hou, Can Rui, Xinyan Wang, Yuru Fan, Ling Zhao, Qing Wang, Zhichong Wang, Xin Zeng, Shanwu Feng, Ping Li

**Affiliations:** ^1^Nanjing Maternity and Child Health Care Hospital, Women’s Hospital of Nanjing Medical University, Nanjing, China; ^2^The Fourth School of Clinical Medicine, Nanjing Medical University, Nanjing, China

**Keywords:** *Candida albican*, vulvovaginal candidiasis, placenta, vascularization, pregnancy outcomes

## Abstract

Vulvovaginal candidiasis (VVC) is considered the second most common cause of vaginitis after bacterial vaginosis and the most common lower genital tract infection during pregnancy. *Candida albicans* (*C. albicans*), an opportunistic pathogen, is the major species causing VVC. Recently, increasing researches have shown that lower reproductive tract infection during pregnancy can lead to various adverse pregnancy outcomes. However, the underlying mechanisms are not fully understood. Hence, we successfully established a mouse model of vaginal *C. albicans* infection and characterized the adverse pregnancy outcomes. *C. albicans* infection strikingly increased abortion rate and decreased litter size. Further analysis of placental development demonstrated that placental structure was abnormal, including that the area of spongiotrophoblast (Spo) and labyrinth (Lab) was reduced, and the formation of placental vessel was decreased in Lab zone. Accordingly, the expression of marker genes during placental development was downregulated. Collectively, the above findings revealed that vaginal *C. albicans* infection during pregnancy can inhibit placental development and ultimately lead to adverse pregnancy outcomes. This study enhances our comprehension of the effect of VVC on pregnancy, and placental dysplasia as a feasible orientation to explore VVC during pregnancy.

## Introduction

Vaginal microbiota plays an important role in maternal and fetal health. Previous studies confirmed that most vaginal microbial communities (73%) were dominated by *Lactobacillus* and a dynamic equilibrium state of coexistence of various microbes in physiological conditions (Stapleton, 2016). Vulvovaginal candidiasis (VVC) is considered the second most common cause of vaginitis after bacterial vaginosis (Gonçalves et al., 2016). Epidemiological studies have shown that 75% of women will experience at least an episode of VVC in their lifetimes, and 40–50% of initially infected women will suffer a relapse (Jaeger et al., 2013; Gonçalves et al., 2016).

*Candida* is the leading species of VVC, and *Candida albicans* (*C. albicans*) is the most common one (90%). *C. albicans* is a conditional pathogen; it is estimated that approximately 10–15% of asymptomatic women are colonized with *Candida* (Gonçalves et al., 2016). The physiological changes of pregnancy are a risk factor for VVC (Aguin and Sobel, 2015). The risk of VVC for non-pregnant women is approximately 20%, but it increases to 30% averagely during pregnancy. Especially in the last trimester, the risk can be as high as 50% (Gonçalves et al., 2016; Sangaré et al., 2018; Konadu et al., 2019; Waikhom et al., 2020). During pregnancy in women, there are increased levels of progesterone and estrogen, which affect the vaginal microenvironment and easily lead to vaginal microbiome disorder with subsequent lower reproductive tract infection (Huang et al., 2014; Greenbaum et al., 2019). Recently, accumulating evidences have demonstrated that the abnormal vaginal microbiota during pregnancy may be associated with the adverse outcomes of mother and child (Donders et al., 2009; Stout et al., 2017). There has been evidence indicating that candidiasis during pregnancy may be related to an increased risk of pregnancy complications, such as preterm birth (PTB), abortion, premature rupture of membranes, and other adverse pregnancy outcomes (Mazor et al., 1993; Ilkit and Guzel, 2011; Roberts et al., 2011; Ali et al., 2012). Roberts et al. (2011) found that, compared with pregnant women without candidiasis, the rate of PTB was higher in asymptomatic candidiasis patients (6.25%: 2.99%). In addition, there is a decreasing trend in the incidence of preterm delivery in antifungal-treated women (Roberts et al., 2011). Particularly, *Candida* infection is the most common vaginal microorganism in patients in the middle and late trimesters of pregnancy, and it can lead to intrauterine infection, endometritis, chorioamnionitis, and neonatal infection as well as PTB and abortion (Aguin and Sobel, 2015).

The adverse pregnancy outcomes caused by VVC in pregnancy have attracted extensive attention in the field of obstetrics and gynecology. However, its mechanism is still unclear. The mechanism of lower genital tract infection leading to adverse pregnancy may consist of ascending infection (Vornhagen et al., 2016; Pavlidis et al., 2020), abnormal immune response (Mor et al., 2011; Helmo et al., 2018), and placental dysfunction (Yockey et al., 2016). Our previous study found that immune response followed by vaginal *Escherichia coli* infection could result in placenta macrophage polarization anomaly and then lead to pregnancy loss in mice (Fan et al., 2021). The placenta, a special temporary organ during the gestation of mammals, forms the maternal–fetal interface and plays a vital role in the health of the mother and the development of the fetus (Ander et al., 2019). Placental development during pregnancy is influenced by numerous factors, such as maternal nutritional status, lifestyle, inflammation, and viral infection. Placental dysplasia can affect the development of the fetus and even result in abortion and stillbirth. Studies have shown that folate deficiency and exposure to physicochemical factors (acrylamide, diphthalate, titanium dioxide) during pregnancy could lead to absorbed embryos and impair the placental development in mice (Zong et al., 2015; Zhang et al., 2018; Yin et al., 2019; Yu et al., 2019). It has been suggested that maternal inflammation during pregnancy leads to placental function defects and fetal growth restriction (FGR). Small-for-gestational-age infants (SGA) are often accompanied by placental deficiency, which is manifested as placental hypoperfusion. However, there have been no studies on whether gestational VVC leads to adverse pregnancy outcomes by affecting placental development.

The placenta derives from the outer trophectoderm layer of the blastocyst in mammals. In mice, at embryonic day (E) 3.5 of development, two distinct cell lineages are formed: the outer specialized trophectodermal epithelium (TE) and the inner cell mass (ICM) (Cross et al., 1994; Rossant and Cross, 2001; Maltepe et al., 2010; Senner and Hemberger, 2010). By E4.5, around the time of blastocyst implantation, the placenta begins to develop (Yan et al., 2001). The ectoplacental cone (EPC, placental primary structure) is fully formed and begins to generate the fetal components of the placental vascular network at around E8.5 (Rossant and Cross, 2001). At E12.5, the placental structure has basically matured, among which the trophoblast cells are roughly divided into three layers from the outside to the inside (maternal side to fetal side): trophoblast gaint cell (TGC), spongiotrophoblast (Spo), and labyrinth (Lab) (Rossant and Cross, 2001; Cross, 2005; Hu and Cross, 2010). All TGCs require the transcription factor heart and neural crest derivatives expressed 1 (Hand1) to regulate their differentiation process (Nakayama et al., 1997; Simmons et al., 2007). The middle layer of the mature placenta is the Spo, a cellular layer that does not move and mainly functions as a support. The formation of this layer depends on the transcription factor achaete-scute family bHLH transcription factor 2 (Ascl1/Mash2) (Guillemot et al., 1995). The Lab, located in the innermost layer of the placenta, is highly vascularized and is a place for maternal–fetal material exchange. The formation of labyrinthous villi originated from the development of the Lab layer; a small cluster of cells in the ectoderm (chorionic plate) begin to express glial cells missing homolog 1 (*Gcm1*) gene around E7.5 (Anson-Cartwright et al., 2000). *Gcm1* gene is only expressed at the top of branching, and these branches are considered as the beginning of syncytiotrophoblast (SynT) differentiation in the Labs (Cross et al., 1994). In addition, extraembryonic spermatogenesis homeobox 1 (Esx1) and fos-like antigen 1 (Fosl1/Fra1) are also involved in the process. Until late gestation (E18.5), the placenta develops completely (Adamson et al., 2002).

This study was designed to establish a vaginal infectious mouse model during pregnancy to evaluate the effect of VVC on placenta development and pregnancy outcome and aimed to test the hypothesis that maternal VVC disrupted post-implantation fetal growth during pregnancy via dysregulation placental development. The results showed that VVC during pregnancy in mice led to an increase in the proportion of resorbed fetuses and the abortion rate of pregnant mice. Compared with the control group, the proportions of EPC and the area of Lab were significantly smaller on E8.5, E11.5, and E18.5, respectively. Meanwhile, the number of blood vessels in the placental Lab layer in the vaginal infection group was reduced. Collectively, the above results provide insight into the exploration of VVC during pregnancy. According to the effect of vaginal *C. albicans* infection on placental development, the abnormal placental structure development may account for adverse pregnancy outcomes resulting from VVC. Our work deepens the understanding of adverse pregnancy resulting from vaginal *C. albicans* infection and explains the phenomenon from the perspective of placental development.

## Materials and Methods

### Reagents and Antibodies

The following antibodies were used during this study: rabbit anti-laminin antibody (ab11575), Cytokerin8 (ab53280), Ki-67 (ab16667), Bax (ab32503), Bcl-2 (ab182858), caspase-8 (ab25901), glyceraldehyde-3-phosphate dehydrogenase (GAPDH) (ab8245), and goat polyclonal Secondary Antibody to Rabbit IgG-H&L (ab150077) were purchased from Abcam (Cambridge, MA, United States). A protease inhibitor cocktail and a phosphatase inhibitor cocktail were obtained from Cell Signaling Technology (Boston, United States). Radioimmunoprecipitation assay (RIPA) was from Beyotime (Shanghai, China). Bicinchoninic acid (BCA) protein assay kit, RNA isolation kit, the reverse transcription kit, and real time SYBR Green Mix were from Vazyme (Nanjing, China).

### Strains and Culture Conditions

*C. albicans* ATCC 64548 used in this research was provided by the Microbiological Lab of Nanjing Drum Tower Hospital (Nanjing, China). It was cryopreserved in 15% glycerinum at-80°C. For preparation of *C. albicans* suspensions, *C. albicans* was melted softly on ice, and then viable *C. albicans* were quantified by 50-fold dilution [series of 10 μl, the yeast in 500 μlLuria–Bertani (LB) medium] of inoculation stocks, shaken at 220 rpm, 37°C for 12 h. Vaginal lavage fluid cultured in Triphenyte-trazoliumchloride (TTC)-Sabouraud medium (SM), sealed with clear adherent sealing film (Corning, United States) then incubated at 37°C for 24 or 48 h. The detailed methods of vaginal lavage fluid culture followed our previous work (Fan et al., 2021).

### Animal Experiment

All specific pathogen-free (SPF) male and female BALB/C mice (6–8 weeks), weighing approximately 20.0 ± 1.0 g, were purchased from Beijing Vital River Laboratory Animal Technology (Beijing, China). The experiment was approved by the Animal Research Ethics Board of Nanjing Medical University (Approval Number: IACUC-2008043). Mice were housed in the SPF laboratory environment in the Animal Care Facility of Nanjing Medical University.

Mice were accustomed for 7–10 days and then mated. The fertile males were copulated in the same cage with virgin females to induce the pregnancy at a ratio of 1:2. The day of finding a vaginal plug was considered as embryonic day 0.5 (E0.5). The pregnant mice were then randomly divided into two groups: the VVC group (*n* = 24) and the control group (*n* = 23). The pregnant female mice received either *C. albicans* in LB medium [20 μl; 2% (v/v); about 2 × 10^7^ CFU] (Conti et al., 2014; Luan et al., 2020) or the same volume of phosphate-buffered saline (PBS) control via intravaginal inoculation at E4.5 in the VVC group and the control group, respectively. Care was taken to minimize vaginal leakage of the administered bolus.

For the vaginal infection model, the following tissues were collected: vaginal lavage fluid (10 μl of PBS was flushed into the vagina right in pregnant mice). The pregnant mice were weighed and sacrificed by cervical dislocation, then rapid cesarean section for vaginas, uterus, fetuses, and placenta collection at E8.5, E11.5, and E18.5, respectively (numbers of mice in the control group: E8.5 *n* = 8, E11.5 *n* = 8, E18.5 *n* = 8; numbers of mice in the VVC group: E8.5 *n* = 7, E11.5 *n* = 8, E18.5 *n* = 8). The uterus, fetuses, and placentas from these mice were photographed. Vaginas, uterus, and placentas were cut in half and then fixed in formaldehyde solution or frozen at -80°C for further analysis separately. The weight of fetuses and the placenta was recorded at E18.5, and the weight of pregnant mice was also documented at E0.5, E4.5, E8.5, E11.5, and E18.5, respectively.

### Hematoxylin-Eosin Staining

The vaginal and placental tissues were fixed in 4% formaldehyde solution and then dehydrated through a series of alcohol baths, cleared in xylene, and dipped in wax after appropriate embedding. Paraffin sections (5-μm thickness) were cut from array blocks and transferred to glass slides (CITOTEST, Jiangsu). Then sections stained with hematoxylin–eosin (H&E) were observed and photographed using a conventional light microscope (or digital slice scanner) for morphological evaluation. At E8.5, the embryonic tissues sections were observed for the development of EPC. At E11.5 and E18.5, the areas of whole placenta, placental Spo, and placental Lab were measured using sections for the layer of whole placenta, using Caseviewer software (v 2.4.0.119028, 3DHISTECH Ltd.). The vaginal tissue sections were used to assess inflammatory condition at different periods.

### Immunohistochemistry

The tissues were fixed in 4% formaldehyde solution, dehydrated, and embedded in paraffin. Tissue sections were mounted on glass slides. And then these sections of the formalin-fixed, paraffin-embedded (FFPE) tissue samples were deparaffinized in xylene and rehydrated in decreasing concentrations of ethanol. The sections were then incubated with 3% v/v H_2_O_2_ for 10 min at room temperature. Non-specific bindings were blocked using 5% v/v bovine serum albumin (BSA) for 30 min at 37°C. Then, the sections were incubated with the corresponding primary antibodies at 4°C overnight; after washing in PBS three times for 5 min each time, the sections were incubated with a secondary antibody (1:200) for 50 min at 37°C. The primary antibody was detected with fresh diaminobenzidine solution, together with counter-staining with hematoxylin. The following primary antibodies were used: CK8 (1:200), laminin (1:200), and Ki-67 (1:250). TdT-mediated dUTP nick end labeling (TUNEL) staining for apoptotic cells was done using paraffin sections following the In Situ Cell Death Detection Kit, POD (Roche, Basel, Switzerland) manufacturer’s protocol. Apoptotic cells were labeled with fluorescein, and sections were counterstained with propidium iodide.

### Isolation of Total RNA and Quantitative PCR

Total RNA was extracted from tissues with RNA isolation kit (Vazyme, China) according to the manufacturer’s instructions. Total RNA was converted into cDNA in a 20 μl reaction mixture following a reverse transcription kit (Vazyme, China). Real-time PCR was then performed in a 10 μl reaction volume containing 5 μl 2 × ChamQ Universal SYBR Green qPCR Master Mix (Vazyme, China), 4 μl of template cDNA, and 0.5 μM primers. PCR protocol included the following steps: 95°C for 30 s; 40 cycles (95°C for 10 s, 60°C for 30 s); 95°C for 15 s; 60°C for 60 s and 95°C for 15 s in Viia7 system. Data were analyzed using the 2^–△^^△^ *^CT^* method with normalization to *Gapdh* expression. The primers used are listed in Supplementary Table 1.

### Western Blot Analysis

The tissues were homogenized in the mixture with RIPA buffer and phenylmethanesulfonyl fluoride (PMSF) (100:1) to extract protein. The protein content was measured with BCA protein assay kit. The proteins were denatured by boiling in 5 × loading buffer, then separated by 10% sodium dodecylsulfate-polyacrylamide gel for electrophoresis (SDS-PAGE) and electroblotted onto a polyvinylidene fluoride (PVDF) membrane. The membrane was blocked for 2 h at room temperature with 5% non-fat dry milk in 1 × tris-buffered saline Tween (TBST). Primary antibodies (1:2,000), Bax (1:2,000), Bcl-2 (1:2,000) and Caspase-8 (1:2,000) were incubated with membranes at 4°C overnight. After washing with TBST three times for 10 min each time, these membranes were then incubated with secondary antibodies at 1:5,000 dilutions at room temperature for 1 h. After washing twice with 1 × TBST for 10 min each time, the immunoreactive proteins were detected using a Tanon 4600 System (Shanghai, China). The relative protein levels were analyzed by Image J software and normalized to Gapdh, which was the internal control.

### Isolation of Microbial Genomic DNA and Agarose Gel Electrophoresis

DNA was extracted from the placental tissues, each sample weighing about 20 mg, using the PowerFecal Pro DNA kit (QIAGEN, Germany) manufacturer’s protocol. The primer sequences showed in Supplementary Material; we used the internal transcribed spacer (ITS) region of the rRNA gene PCR amplification for placentas. The PCR reaction mixture consisted of 10 μl 2 × Taq Master Mix (Vazyme, China), 0.8 μl forward primer (10 μM), 0.8 μl reverse primer (10 μM), 6.4 μl ddH_2_O, and 2 μl template DNA at 95°C for 15 s, 60°C for 60 s, and 95°C for 15 s. The mixture was then prepared for PCR at 95°C for 5 s, then subjected to 40 cycles of denaturation at 95°C for 30 s, annealing at 60°C for 30 s, and extension at 72°C for 2 min, with an additional 10 min extension at 72°C. Products were followed by running on a 1% agarose gel for imaging.

### Statistical Analysis

The data were presented as the mean ± standard deviation (SD). Student’s *t*-test was used to analyze the significant differences between the two groups. Fisher’s exact test was performed on the count data to test the difference in ratios. The differences were considered statistically significant at *p < 0.05, **p < 0.01, and ***p < 0.001. All statistical analyses were performed using GraphPad Prime 8.0 (GraphPad Software, San Diego, CA, United States) or SPSS version 25.0 (SPSS Inc., Chicago, IL, United States).

## Results

### A Model of Vaginal *Candida albicans* Infection in Mouse Pregnancy

**FIGURE 1:**
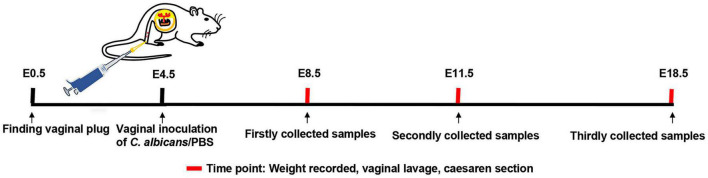
Establishment of a model of vaginal infection with *C. albicans*. Male and female mice were mated, and the day of finding a vaginal plug was considered as embryonic day 0.5 (E0.5). Pregnant mice received *C. albicans* (the VVC group) or PBS (the control group) via vaginal inoculation at E4.5. Then, the weight of mice was recorded, and vaginal flush was carried out at E8.5, E11.5, and E18.5, respectively. Finally, the required tissues were collected by cesarean section at the same time.

After finding vaginal plugs (E0.5), live *C. albicans* or PBS were inoculated into mouse vaginas at E4.5 in the VVC group and control group (Figure 1). Subsequently, vaginal lavage fluid was collected and cultured at E8.5, E11.5, and E18.5. The results are displayed in Supplementary Figure 1A. All colonies were observed on the TTC-SM in the VVC group at different times. The number of colonies decreased gradually as time went on, which probably resulted from vaginal self-purification. However, there was no fungal colony formation in the control group (Supplementary Figure 1A). Then, H&E-stained vaginal tissue was examined (Supplementary Figure 1B). In the VVC group, the vaginal epithelium is infiltrated with inflammatory cells, and even lymphatic follicles were visible. Moreover, the organization of vaginal epithelia was aberrant in the VVC group. All of these results indicated that mice exposed to *C. albicans* suffered from typical vaginal infection.

### Vaginal *Candida albicans* Infection During Early Pregnancy Resulted in Adverse Pregnancy Outcomes

**FIGURE 2:**
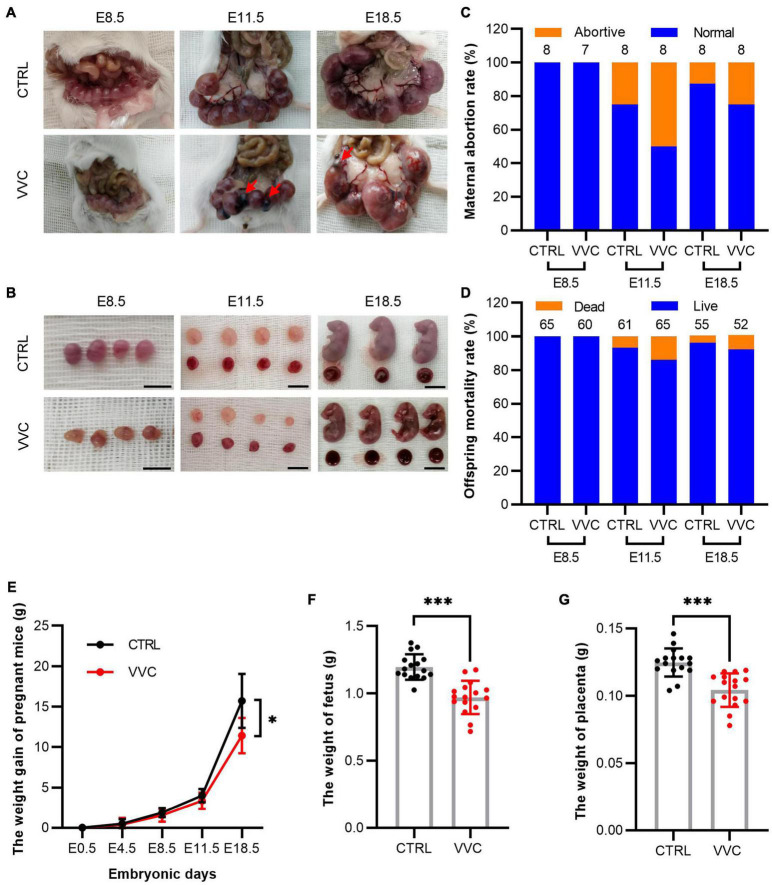
Characteristics of pregnancy outcomes in the VVC group and the control group. **(A,B)** Representative images of embryos and placentas by integral view **(A)** and local view **(B)** at E8.5, E11.5, and E18.5, respectively. Red arrows indicate resorbed embryos. Scale bar: 1.0 cm. **(C,D)** The maternal abortion rate **(C)** and the offspring mortality rate **(D)** in the VVC group and control group at E8.5, E11.5, and E18.5. Numbers of mice in the control group: E8.5 *n* = 8, E11.5 *n* = 8, E18.5 *n* = 8. Numbers of mice in the VVC group: E8.5 *n* = 7, E11.5 *n* = 8, E18.5 *n* = 8. **(E)** Weight recorded of pregnant mice in the VVC group and control group at E0.5, E4.5, E8.5, E11.5, and E18.5. Numbers of mice in the control group: E4.5 *n* = 24, E8.5 *n* = 24, E11.5 *n* = 16, E18.5 *n* = 8. Numbers of mice in the VVC group: E4.5 *n* = 23 E8.5 *n* = 23, E11.5 *n* = 15, E18.5 *n* = 8. **(F)** Weight of fetus in the VVC group and control group by cesarean section at E18.5 (*n* = 16). **(G)** Weight of the placenta in the VVC group and control group by cesarean section at E18.5 (*n* = 16). Data are presented as mean ± SD. Unpaired *t*-test was used for two-group comparisons of the pregnant mouse bodyweight, the weight of fetus, and the weight of the placenta. Fisher’s exact test was used for two-group comparisons of the maternal abortion rate and the offspring mortality rate. *p < 0.05, ***p < 0.001 for VVC vs. CTRL.

According to gestation and embryo staging, at E4.5, the blastocyst is implanted and then divided into early gestation (E8.5, gastrula formation), mid-gestation (E11.5, limbs development), and late gestation (E18.5, placental mature). The numbers of fetus and placenta in each mouse were specifically observed and recorded after cesarean section at different stages. Images suggested that no abortion was found in either group at E8.5, but abortions occurred from E11.5. Meanwhile, the embryos looked smaller than the embryos in the control group at E8.5. At E11.5 and E18.5, the size of fetus and placenta was smaller than that in the control group (Figures 2A,B). To further analyze pregnancy outcomes between the two groups, the abortive mice and resorbed embryos at different times were recorded. The maternal abortion rate was twice as high as in the control group, and the offspring mortality rate in the control group was 6.56% at E11.5 and 4.17% at E18.5, simultaneously, 13.85% at E11.5, and 8.51% at E18.5 in the VVC group (Figures 2C,D). The details of fetus survival of each pregnant mouse are shown in Supplementary Table 2. The maternal bodyweight was recorded at E4.5, E8.5, E11.5, and E18.5 (Figure 2E). The mean increased weight of pregnant mice at E4.5, E8.5, E11.5, and E18.5 in the control group was 0.55 ± 0.56, 1.88 ± 0.59, 3.95 ± 0.83, and 15.65 ± 3.36 g, respectively. The corresponding weight value in the VVC group was 0.41 ± 0.81, 1.56 ± 0.78, 3.35 ± 0.96, and 11.43 ± 2.22 g, respectively. There are significant differences in the maternal weight gain between the two groups at E18.5 (*p* = 0.02). In addition, the mean weight of fetuses (Figure 2F) at E18.5 in the control group (1.20 ± 0.09 g) was higher than that in the VVC group (0.97 ± 0.12 g, *p* < 0.001). Similarly, the weight of the placenta (Figure 2G) was higher in the control group (0.12 ± 0.01 g) than that in the VVC group (0.10 ± 0.01 g, *p* = 0.018, *p* < 0.001). These results suggested that vaginal *C. albicans* infection led to adverse pregnancy outcomes, including abortion and weight reduction of maternal and fetal mice. Notably, placental weight decreased during pregnancy.

### Vaginal *Candida albicans* Infection Impaired Development of Placenta in Mice

**FIGURE 3:**
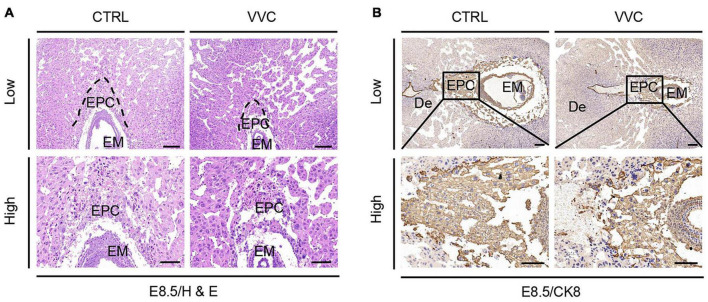
Effect of vaginal *C. albicans* infection on EPC. **(A)** Embryonic tissue was stained using H&E for ectoplacental cones from mice at E8.5. **(B)** Trophoblast cells in ectoplacental cone marked by immunohistochemical staining of CK8 at E8.5. Low magnification scale bar = 200 μm, high magnification scale bar = 100 μm. De, decidua; EPC, ectoplacental cones; EM, embryo.

**FIGURE 4:**
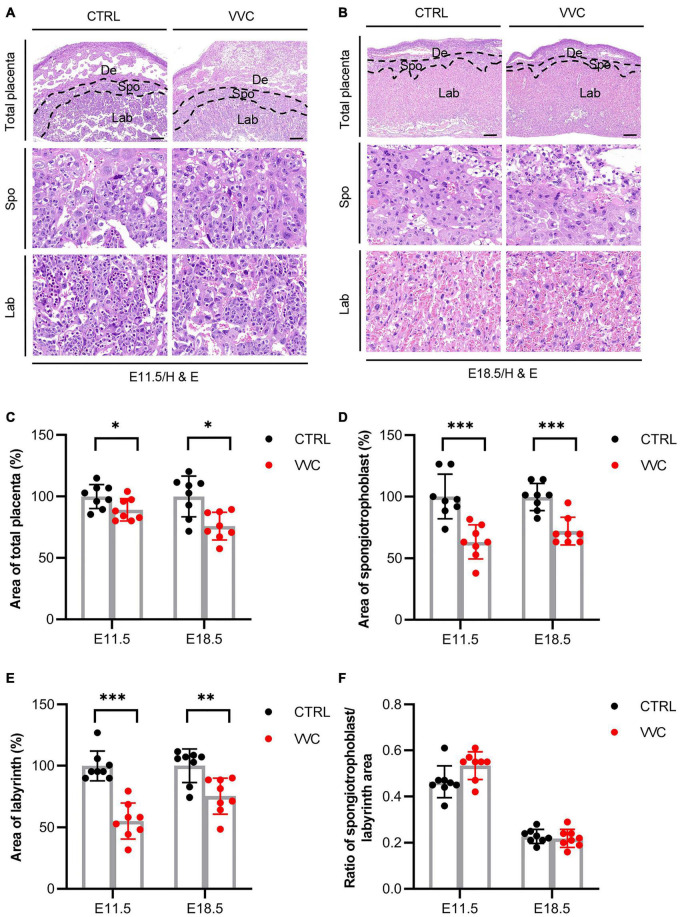
Effect of Vaginal *C. albicans* infection on placental structure. **(A,B)** Representative images of placental section observed by H&E staining at E11.5 **(A)** and E18.5 **(B)**. Scale bar = 200 μm. De, decidua; Spo, spongiotrophoblast; Lab, labyrinth. **(C–F)** Total area of placenta (%) **(C)**, area of spongiotrophoblast (%) **(D)**, area of labyrinth (%) **(E)**, and the ratio of spongiotrophoblast to labyrinth area (%) **(F)** in placental tissue at E11.5 and E18.5. Data are presented as mean ± SD (*n* = 8/group). Unpaired *t*-test was used for two-group comparisons. *p < 0.05, **p < 0.01, ***p < 0.001 for VVC vs. CTRL.

H&E results showed that vaginal *C. albicans* infection significantly inhibited the growth and development of EPC. The proportions of EPC were significantly smaller in the *C. albicans*-infected mice than in the control group at E8.5 (Figure 3A). The immunohistochemical staining of CK8 (a marker of trophoblast cells in EPC) indicated that EPC was atrophic in the VVC group at E8.5 (Figure 3B). This was consistent with the H&E result. A mature placenta consists of the well-defined three layers of maternal decidual (De) layer, Spo layer, and Lab layer. Histological analysis revealed that the area of Spo and Lab had abnormal changes (Figures 4A,B). At E11.5, the area of total placenta, Spo, and Lab had reduced to 89.12, 63.22, and 55.22%. At E18.5, the area of total placenta, Spo, and Lab had reduced to 75.88, 71.99, and 75.36%. However, the ratio of Spo/Lab area showed no significant difference between the two groups (Figures 4C–F). These results indicate that vaginal *C. albicans* infection will impair the development and function of the placenta, and abnormal placenta may be the cause of abortion and fetal weight loss following vaginal *C. albicans* infection.

To investigate the presence or absence of *C. albicans* in placental tissues from vaginal infection during pregnancy, we used ITS region of the rRNA gene PCR amplification to search for molecular markers of fungus as described in section “Materials and Methods”. The result showed that no fungal ITS region gene sequence amplicons were identified by agarose gel electrophoresis in DNA extracted from three selected placentas (Supplementary Figure 2). Thus, there was no colonization of *C. albicans* in placentas with vaginal *C. albicans* infection.

### Effect of Vaginal *Candida albicans* Infection on the Proliferation and Apoptosis of Placenta

**FIGURE 5:**
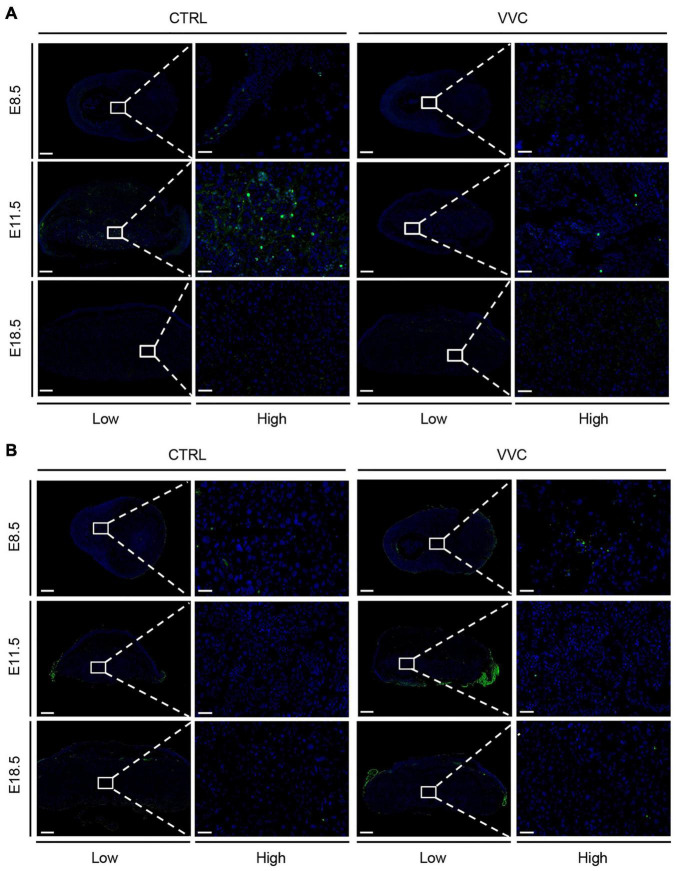
Effect of vaginal *C. albicans* infection on the proliferation or apoptosis of placenta. **(A)** Proliferation of placental trophoblast was assessed by Ki67 staining at E8.5, E11.5, and E18.5. Five placentas per genotype were evaluated. Blue, DAPI; green, Ki67. **(B)** Apoptosis of placental trophoblast was assessed by TUNEL at E8.5, E11.5, and E18.5. Three placentas per genotype were evaluated. Blue, DAPI; green, TUNEL. Ectoplacental cones (E8.5) and placental labyrinth zones (E11.5 and E18.5) are presented at higher magnification. Low magnification scale bar = 500 μm, high magnification scale bar = 50 μm. Representative images are shown.

To explore whether the placental abnormal development in the VVC group was related to the proliferation and apoptosis changes of the placenta, we examined Ki67- and TUNEL-stained placenta and analyzed protein levels of key apoptotic proteins (Bax, Caspase8, and anti-apoptotic proteins Bcl-2). There were no obvious proliferative cells between the two groups at E8.5 and E18.5. However, the number of Ki67-positive cells in the VVC group was lower than that in the control group at E11.5 (Figure 5A). TUNEL staining was indistinguishable between the two groups (at E11.5 and E18.5, this non-specific staining occurred on the far left and right sides of the placenta in the VVC group due to technical errors—partial residual membranes on the surface of placental tissue) (Figure 5B). The number of Ki67-positive cells and dead cells in each group are shown in Supplementary Figures 3A,B. Western blotting results showed that there were no significant differences in the protein levels of Bax, Caspase8, and Bcl-2 between the two groups at different periods during pregnancy (Supplementary Figure 3C).

### Effect of Vaginal *Candida albicans* Infection on Placental Development Genes Expression

**FIGURE 6:**
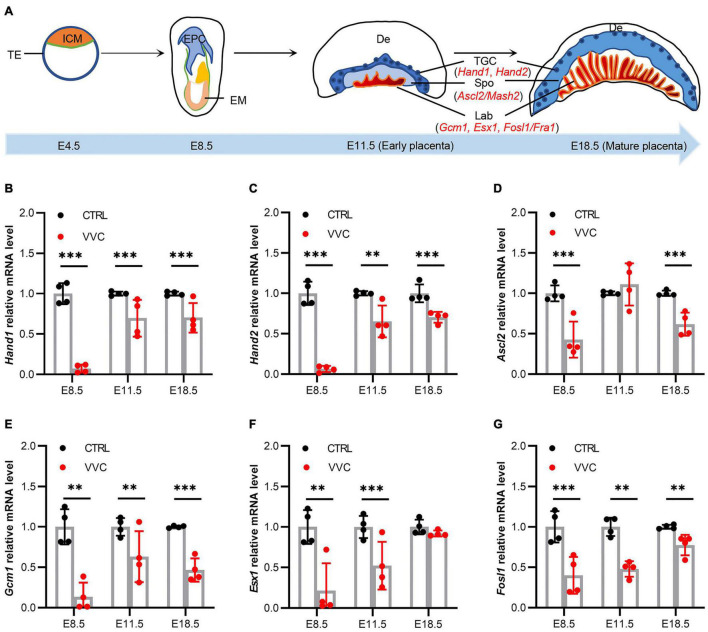
Effects of Vaginal *C. albicans* infection on the expression of placental development genes. **(A)** The schematic diagram of mouse placenta structure. ICM, inner cell mass; TE, trophectoderm; EPC, ectoplacental cones; EM, embryo; De, decidua; TGC, trophoblast gaint cell; Spo, spongiotrophoblast; Lab, labyrinth. Hand1, heart and neural crest derivatives expressed 1; Hand2, heart and neural crest derivatives expressed 2; Ascl2, achaete-scute family bHLH transcription factor 2; Gcm1, glial cells missing homolog 1; Esx1, extraembryonic spermatogenesis homeobox 1; Fosl1, fos-like antigen 1. **(B–G)** Relative expression levels of *Hand1* **(B)**, *Hand2* **(C)**, *Ascl2* **(D)**, *Gcm1* **(E)**, *Esx1* **(F)**, and *Fosl1* mRNA **(G)** in mice placentas from the VVC group and control group at E8.5, E11.5, and E18.5. mRNA levels were quantified using reverse transcription-quantitative polymerase chain reaction (qRT-PCR) and normalized to *Gapdh*. Four samples of each group were used. The experiment was performed in triplicate wells and repeated three times. Data are presented as means ± SD. Unpaired *t*-test was used for two-group comparisons. *p < 0.05, **p < 0.01, ***p < 0.001 for VVC vs. CTRL.

To investigate underlying mechanisms of placental abnormal development in mice following vaginal *C. albicans* infection, we studied the effect of vaginal *C. albicans* infection on marker genes expression in placental development as shown in the diagram (Figure 6A). *Hand1*, *Hand2*, *Ascl2*, *Gcm1*, *Esx1*, and *Fosl1* mRNA expression all exhibited significant downregulation in the placenta tissues of the VVC group compared with that of the control group at E8.5, and the fold change in the VVC group dropped to 7.15, 6.25, 42.63, 13.48, 21.51, and 39.91%, respectively. At E11.5, the trends in the significant gene expression were consistent with those at E8.5 except *Ascl2* gene increased by 11.18% (no significance), and the fold change in the VVC group reduced to 69.45, 65.16, 63.05, 52.22, and 47.76%, respectively. At E18.5, the fold change in the VVC group reduced to 70.05, 70.35, 61.75, 46.67, 89.99 (no significance), and 72.29%, respectively (Figures 6B–G).

### Vaginal *Candida albicans* Infection Disrupted Labyrinth Vascularization of Placentas

**FIGURE 7:**
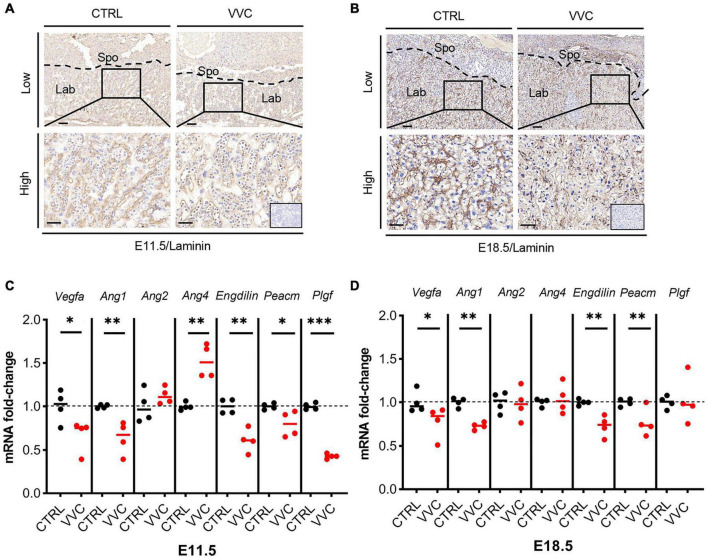
Effect of vaginal *C. albicans* infection on the labyrinth vascularization of placenta. **(A,B)** Laminin immunohistochemical staining of mice placentas from the VVC group and control group at E11.5 **(A)** and E18.5 **(B)**. Low magnification scale bar = 100 μm, high magnification scale bar = 50 μm. Inserted image shows immunostaining of a negative control. Spo, spongiotrophoblast; Lab, labyrinth. **(C,D)** Relative expression of the angiogenesis-related genes (*Vegfa*, *Ang1*, *Ang2*, and *Ang4*), vascular endothelial cell markers (*Endoglin* and *Pecam*), and *Plgf* in the placenta from the VVC group and control group at E11.5 **(C)** and E18.5 **(D)**. Four samples of each group were used. The experiment was performed in triplicate wells and repeated three times. Data are presented as mean ± SD. Unpaired *t*-test was used for two-group comparisons. *p < 0.05, **p < 0.01, ***p < 0.001 for VVC vs. CTRL.

According to the above results that the related genes of Lab zone (*Gcm1*, *Esx1*, *Fosl1*) all exhibited downregulation, we observed placental angiogenesis. Immunohistochemistry staining of laminin (a fetal vessels marker) showed that at E11.5 and E18.5, the number of blood vessels in the placental Lab layer in the VVC group was reduced compared with the control group. Furthermore, the formation of branched fetal vessels was more dramatically decreased than that in the control group, which resulted in fetal blood vessel collapse (Figures 7A,B). Moreover, the expression levels of the angiogenesis-related genes (*Vegfa*, *Ang1*, *Ang2*, and *Ang4*) and vascular endothelial cell markers (*Endoglin* and *Pecam*) were examined. *Vegfa*, *Ang1*, *Endoglin*, and *Pecam* mRNA expression all showed significant downregulation in the placenta tissues of the VVC group compared with that of the control group at E11.5, and the fold change in the VVC group dropped to 66.95, 63.90, 61.10, and 79.78%, respectively (Figure 7C). Similarity, at E18.5, the corresponding gene reduced to 77.58, 72.88, 72.54, and 77.02%, respectively (Figure 7D). At E11.5, the mRNA level of *Ang4* increased by 52.45% compared to the control group, whereas there was no obvious change at E18.5. There was no significant difference in the expression of *Ang2* mRNA between the two groups at E11.5 and E18.5. Placental growth factor (Plgf), an angiogenic factor which belongs to vascular endothelial growth factor (Vegf) family, was downregulated in the placenta of the VVC group at E11.5 (reduced to 42.96%). However, at E18.5, there was no obvious change in the expression of *Plgf* mRNA between the two groups (Figures 7C,D). These results show that vaginal *C. albicans* infection during early pregnancy would impair vascularization in placental Lab, which may be detrimental to the pregnancy process.

## Discussion

The vaginal microbiome is closely related to pregnancy outcome. VVC caused by *C. albicans* is the most common vaginitis during pregnancy. Accumulating studies have confirmed that gestational VVC can lead to premature delivery, abortion, premature rupture of membranes, and other adverse pregnancy outcomes (Mazor et al., 1993; Ilkit and Guzel, 2011; Ali et al., 2012). The present study is, to the best of our knowledge, the first comprehensive investigation to examine the effects of vaginal *C. albicans* infection on mouse pregnancy *in vivo*. Our results showed that *C. albicans* infection significantly increases the abortion rate and decreases the litter size on mid-gestation and late gestation in mice. Moreover, *C. albicans* exposure reduced the bodyweight of mature fetuses before parturition. In addition, Placental development could be inhibited by *C. albicans* infection, mainly manifested as abnormal placental structure, the reduction of placental vessels, the downregulation of placental marker genes, and the suppression of proliferation on placental trophoblast. These findings suggest that adverse pregnancy outcomes including low birth weight and pregnancy loss followed by vaginal *C. albicans* infection are possibly mediated, at least in part, via the suppression of placental growth and development.

In mice, E4.5 corresponds to the initiation of embryonic development at the late blastocyst stage, then E8.5 corresponds to early gestation and the beginning of organogenesis, E11.5 corresponds to mid-gestation and morphogenesis, and E18.5 corresponds to late gestation and just prior to birth (Ko, 2001). Our results indicated that there was no statistic difference in the pregnancy outcome between the control and VVC groups at E8.5. However, the mortality of offspring and the abortion of mice increased significantly at E11.5 and E18.5 in the VVC group, which probably contributes to the time it takes for vaginal infections to work. The consequences demonstrated that VVC during pregnancy can lead to adverse pregnancy outcomes. In addition, we found that vaginal infection with *C. albicans* in pregnancy could cause weight loss in the mother and fetus. Therefore, it is suggested that *C. albicans* manifests toxicity principally toward the growth and development of the fetus.

Cell lineage studies evidenced that the placental and extraembryonic membranes are largely derived from the trophoblast. At E8–9, the polar trophectoderm cells form a cap cone-shaped structure, known as the EPC. The EPC is placental primordial tissue, which will develop the whole placenta (Cross, 2000). Around E12, the placenta is initially mature and divided into four layers, including decidua, trophoblast giant cell, Spo, and Lab (Oh-McGinnis et al., 2011). At late gestation (E18.5), the placenta completely matures, which is characterized by being highly vascularized in the Lab zone. Therefore, we chose E8.5, E11.5, and E18.5 to observe the effect of *C. albicans* exposure on the development of placenta. The results indicated that *C. albicans* infection inhibited the growth and development of the EPC at E8.5, which could result in a placental development disorder. It is demonstrated that placental development is regulated by multiple genes. Hand1 and Hand2 are located in trophoblast cells and promote the differentiation of trophoblast giant cells (Scott et al., 2000; Tsuchihashi et al., 2011). Ascl2 is essential to Spo maintenance and the development of trophoblast lineage (Oh-McGinnis et al., 2011). Gcm1 is required for mouse labyrinthine development and mesoderm formation (Li and Behringer, 1998; Anson-Cartwright et al., 2000). In addition, Esx1 and Fosl1 also play a crucial role in the labyrinthine development vascularization at the maternal–fetal interface (Li and Behringer, 1998; Schreiber et al., 2000). Our results showed that the expression levels of *Hand1* and *Hand2* significantly decreased in the VVC group at E8.5 and E11.5, which probably led to retardation in the differentiation of trophoblast cells and placental growth. Expression levels of *Ascl2* also markedly decreased, which suggested the development of Spo had been affected to some extent. Moreover, downregulation of *Gcm1*, *Esx1*, and *Fosl1* expression would severely impair the development of Lab and result in the reduction of Lab area in the VVC group. These changes ultimately have adverse effects on the development of placenta.

Vascularization of placental Lab is essential for the growth and development of the placenta, and maternal–fetal interface blood supply is vital to maintain successful pregnancy. Vaginal *C. albicans* infection inhibited the formation of fetal vessels and reduced the number of fetal blood vessels in the placental Lab, mainly manifested as placental vascular collapse, and impaired the formation of an intricate network of fetal vessels, which may lead to the development disorder of placenta and fetus. However, its potential mechanism is still poorly understood. Previous research indicated that *Gcm1*, *Esx1*, and *Fosl1* were closely related to placental vascularization. In our study, the level of these genes mRNA showed a consistent downregulation in the VVC group, which possibly contributed to the decrease of fetal vessels within the placental Lab. Numerous signaling molecules, receptors, and signaling transduction pathways regulate angiogenesis, in which the Vegf family and the angiopoietin (Ang) family are predominant. The Vegf family can initiate vascular formation, and Vegfa plays a representative function. The Ang family consists of four members which are considered to play the angiogenesis role at a later time points (Gale et al., 2002; Nagashima et al., 2013). Ang1, as a vessel-stabilizing factor, is complementary to Vegfa during early vascular development, which can promote vascular remodeling, maturation, and stabilization (Gale et al., 2002), whereas Ang2 as a permeability and vessel branching factor plays partial antagonism against Ang1. Ang4 plays a role in the vessel endothelial cells migration and further promotes vascular maturation (Gale et al., 2002). Both Ang1 and Ang4 can activate tek receptor tyrosine kinase (Tie-2) receptor to promote vessel maturation. Our quantitative PCR results showed the downregulation of *Vegfa* and *Ang1* mRNA levels in placentas from the VVC group. However, the Ang1 and Ang 4 levels of mRNA expression showed an opposite trend at E11.5. we speculated that VVC may affect the initiation stage of vascular development, and Ang4 upregulation may be a compensatory consequence of Ang1 downregulation. Endoglin (CD105) and Pecam (CD31) as markers are expressed in vascular endothelial cells. The downregulation of *Endoglin* and *Pecam* mRNA levels in the VVC group also indicated the reduction of the number of placental vessels. The placenta growth factor (Plgf) is an angiogenic factor, which is derived from the placenta and belongs to the Vegf family. It has been considered as a crucial factor for successful pregnancy (Kridli et al., 2016). Plgf exerts its functions via fms-like tyrosine kinase 1 (Flt-1) receptor to reinforce angiogenesis (Nejabati et al., 2017), and studies have also indicated that Plgf may accelerate placental villous proliferation (Athanassiades and Lala, 1998). Similar to the above result, the mRNA expression level of *Plgf* was downregulated as well. These results suggested that *C. albicans* exposure may inhibit vascular development at E11.5, especially at the beginning of vascular formation during the development of placenta. The confirmation of its underlying mechanism requires further investigation.

We also observed whether defective placental development and pregnancy loss caused by exposure to *C. albicans* were associated with the proliferation and apoptosis changes of placenta. Ki67 and TUNEL staining were used to assess proliferation and cell death between the two groups. Ki67-positive cells were downregulated in the VVC group at E11.5, whereas it was not obvious between the two groups at E8.5 and E18.5. Meanwhile, there is no significant difference in cell death. In concordance with TUNEL staining, Western blotting analysis suggested that the protein level of apoptosis-related genes (*Bax*, *Bcl-2*, *Casp-8*) showed no notable change between the two groups. These results suggested that *C. albicans* exposure could inhibit the proliferation, yet it did not affect the apoptosis of placental trophoblast. According to the placental histology analysis, we speculate that the atrophy of EPC and the reduction of placental area could result from the decreased number of trophoblast cell. At E11.5, the primary placenta forms, and the placental blood vessels begin to branch. The low proliferation of placental cells at this time may have an influence on the formation of the placental blood vessel network except affecting placental cell density and function. In combination with the previous results of placental developmental markers, vaginal *C. albicans* infection could also inhibit trophoblast cells differentiation, and furthermore, the function of trophoblast cells may be affected. Therefore, further work is needed to completely elucidate the mechanisms of how vaginal *C. albicans* infection inhibits trophoblast differentiation in mice.

The mechanism of vaginal infection leading to adverse pregnancy has not been elucidated completely. Up to now, it is believed that ascending infection could play an important role in adverse pregnancy outcomes. Ascending infection by microorganisms (trichomonad, bacteria, fungi, etc.) of the lower genital tract (vagina or cervix) is the most common route of intrauterine infection. It is well known that intrauterine infection is a critical factor leading to adverse pregnancy. Research demonstrated that intrauterine infection can lead to abortion in early gestation. Meanwhile, it can also give rise to FGR, premature rupture of membranes, premature delivery, congenital malformation, neonatal infection in mid-gestation, and late gestation (Burd et al., 2012; Shcherbina and Vygivska, 2018). Traditionally, the fetus lies in a sterile environment. Recently, several studies have found the existence of bacteria or microbial DNA in placentas (Offenbacher et al., 1998; Aagaard et al., 2014). However, the latest research found no convincing evidence for the existence of the fetoplacental microbiome (Kuperman et al., 2020). Our results showed that there was no colonization by *C. albicans* in placentas from mice that received the fungus via intravaginal inoculation in early pregnancy. In addition, our results indicated that vaginal infection can affect placental angiogenesis, which may be one of the factors leading to abortion. However, the underlying mechanisms are still unclear. Therefore, further investigation is required to confirm and explain its mechanism.

Collectively, these data provide evidence that vaginal *C. albicans* infection significantly inhibited the growth and development of placenta through systemic regulation rather than ascending infection, which probably results in adverse pregnancy outcomes including high pregnancy loss, low pregnancy rates, and low birth weight in pregnant mice exposed to *C. albicans*. Furthermore, our results showed that retarded placental development in *C. albicans*-infected mice was associated with some aspects involved in the dysregulation of placental development marker expression and the formation of Lab vessels. The innovation of this study is that we initially confirmed abnormal placental development for the adverse pregnancy outcomes resulting from vaginal *C. albicans* infection. Therefore, placental development can be considered as a research point for adverse pregnancy caused by vaginal *C. albicans* infection. However, further research efforts are required to completely elucidate the mechanism of vaginal infection-induced compromised placental development.

## Data Availability Statement

The original contributions presented in the study are included in the article/Supplementary Material, further inquiries can be directed to the corresponding author/s.

## Ethics Statement

The animal study was reviewed and approved by the Animal Research Ethics Board of Nanjing Medical University.

## Author Contributions

ZD, CF, WH, and XZ designed the experiments. ZD, CF, WH, LZ, QW, and ZW carried out experiments and performed data analysis. CR, XW, and YF provided scientific advice. ZD and XZ wrote the manuscript. XZ, SF, and PL supervised the project and also offered funding support. All authors contributed to the article and approved the submitted version.

## Conflict of Interest

The authors declare that the research was conducted in the absence of any commercial or financial relationships that could be construed as a potential conflict of interest.

## Publisher’s Note

All claims expressed in this article are solely those of the authors and do not necessarily represent those of their affiliated organizations, or those of the publisher, the editors and the reviewers. Any product that may be evaluated in this article, or claim that may be made by its manufacturer, is not guaranteed or endorsed by the publisher.
