# Supplementary material for: Vaginal Exposure to Candida albicans During Early Gestation Results in Adverse Pregnancy Outcomes via Inhibiting Placental Development
Source: Front Microbiol. 2022 Feb 24;12:816161. doi: 10.3389/fmicb.2021.816161 (PMC8908259; doi:10.3389/fmicb.2021.816161)
Supplement: Supplementary file 1 [file Data_Sheet_1.docx]

Supplementary Material

# Supplementary Tables

**Table 1.** Primer sequences for real-time PCR

| Gene |  | Primer sequence (5'-3') |
| --- | --- | --- |
| *Gapdh* | Forward | AAGAAGGTGGTGAAGCAGG |
|  | Reverse | GAAGGTGGAAGAGTGGGAGT |
| *Hand1* | Forward | GCTGATGCTGCCCCAGATTTC |
|  | Reverse | GGGTCCTGAGCCTTTTCGTTT |
| *Hand2* | Forward | TCGTTGCCTACAGAAACCTTCA |
|  | Reverse | CGAGCAAGGCTGGAGATGAC |
| *Ascl2* | Forward | TGGCACGCCGCAATG |
|  | Reverse | CCTGGAAGCCCAAGTTTACCA |
| *Gcm1* | Forward | ATGGCGGGTCTCAGGTAAATC |
|  | Reverse | CGCAACACCAACAACCACAAC |
| *Esx1* | Forward | TTTTCCAGCGCGTCCAGTA |
|  | Reverse | TCGGGCAAGCTCCACTCT |
| *Fosl1* | Forward | ACCTTGTGCCAAGCATCGA |
|  | Reverse | AATGAGGCTGCACCATCCA |
| *Vegfa* | Forward | TGCTGGCTTTGGTGAGGT |
|  | Reverse | TACTGCCGTCCGATTGAGA |
| *Ang1* | Forward | GGAACCGAGCCTACTCACAG |
|  | Reverse | CAAGCTGCTCTGTTTGCCTG |
| *Ang2* | Forward | TCATCTGGAGAAGCACACATTCA |
|  | Reverse | ACAGTCTCCGCATTCACCAACA |
| *Ang4* | Forward | GAAGCTGGAGCAGTCCATCA |
|  | Reverse | TTCATGAGGTTGGCACCCAG |
| *Endoglin* | Forward | CCCTCTGCCCATTACCCTG |
|  | Reverse | GTAAACGTCACCTCACCCCTT |
| *Pecam* | Forward | CTGCCAGTCCGAAAATGGAAC |
|  | Reverse | CTTCATCCACCGGGGCTATC |
| *Plgf* | Forward | CATAGTGATGTTGGCTGTCTTT |
|  | Reverse | GTCCTTCTGAGTCGCTGTAGTG |
| Fungal ITS primer1 | Forward | TTTATCAACTTGTCACACCAGA |
|  | Reverse | AATGAGGCTGCACCATCCA |
| Fungal ITS primer2 | Forward | TGGTAAGGCGGGATCGCTT |
|  | Reverse | GGTCAAAGTTTGAAGATATAC |
| Fungal ITS primer3 | Forward | GCATCGATGAAGAACGCAGC |
|  | Reverse | TCCTCCGCTTATTGATATGC |

**Table2.** The detail of pregnancy outcome in mice between the two group.

| Group | Control  Live + Dead fetus | n | Abortion rate | VVC  Live +Dead fetus | n | Abortion rate |
| --- | --- | --- | --- | --- | --- | --- |
| E8.5 | 8+0, 9+0, 8+0, 5+0, 9+0, 8+0, 8+0, 10+0 | 8 | 0/8 | 9+0, 8+0, 9+0, 8+0, 8+0, 9+0, 9+0 | 7 | 0/7 |
| E11.5 | 9+0, 9+0, 7+2, 6+0, 6+2, 8+0, 7+0, 5+0 | 8 | 2/8 | 3+3, 8+0, 7+1, 6+3, 9+0, 7+0, 8+2, 8+0 | 8 | 4/8 |
| E18.5 | 7+0, 3+0, 8+0, 7+0, 9+0, 6+0, 6+2, 7+0 | 8 | 1/8 | 5+3, 7+1, 1+0, 5+0, 9+0, 8+0, 8+0, 5+0 | 8 | 2/8 |

Supplementary Figures


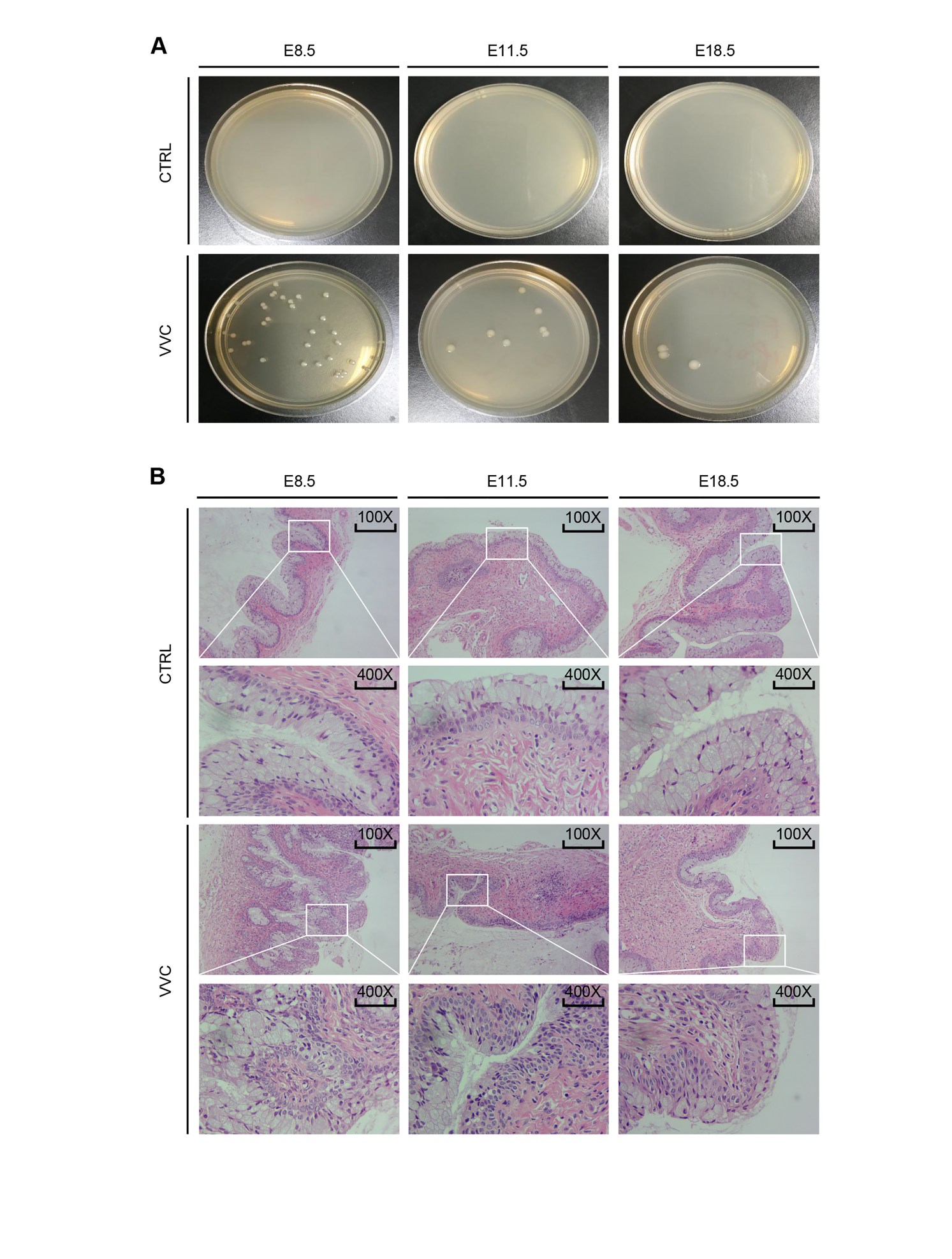


**Supplementary Figure 1.** Establishment of a model of vaginal infection with C. albicans.

**(A)** Representative images of vaginal lavage fluid culture on TTC-Sabouraud medium at E8.5, E11.5, and E18.5, respectively. Colonies all were exhibited in the VVC group, and the colony count decreased gradually as time went on.

**(B)** H&E staining of vaginal tissue in the VVC group and the control group of mice at E8.5, E11.5, and E18.5. Showing neutrophil infiltration in the VVC groups. The magnification of the microscope examination used was 100× and 400×.


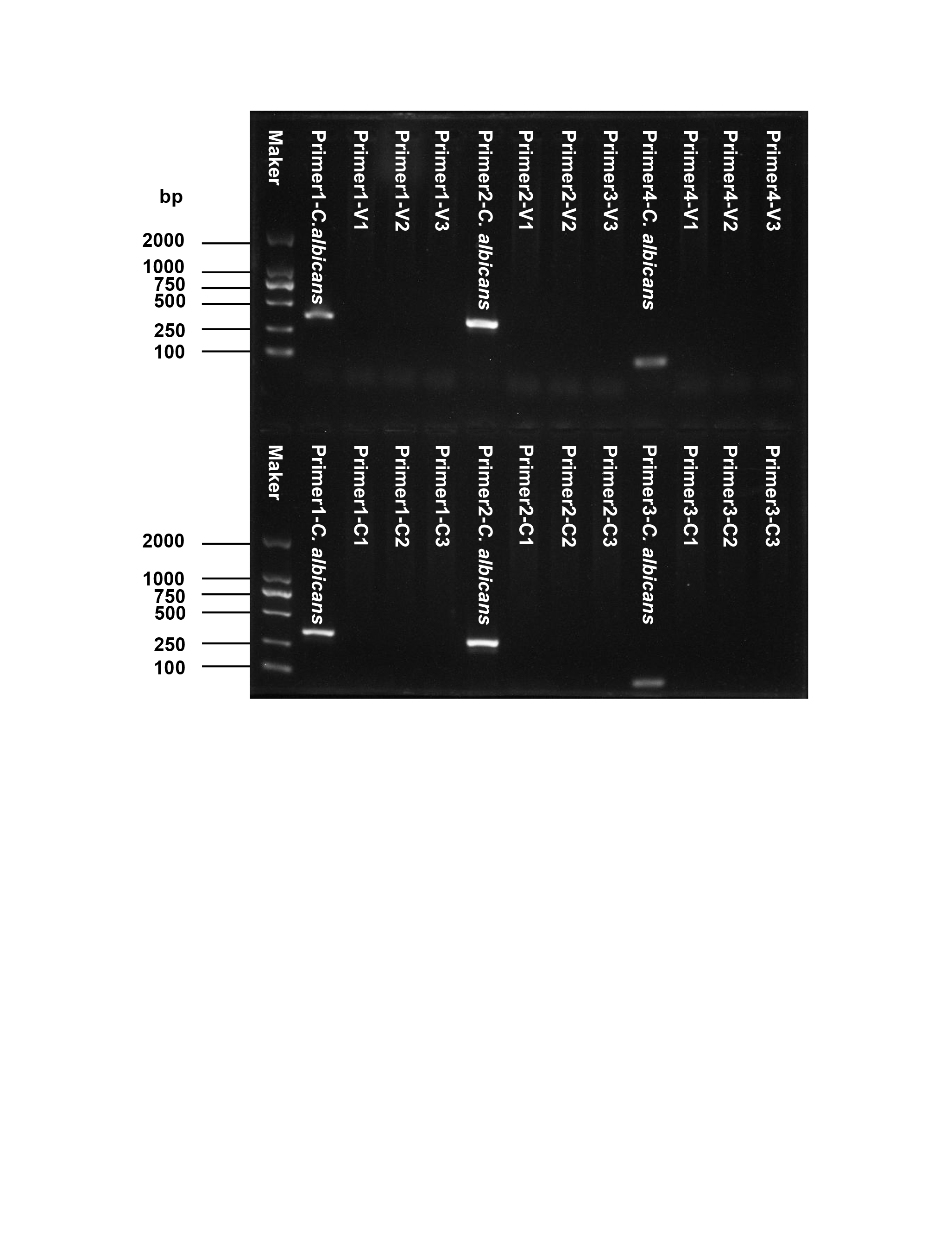


**Supplementary Figure 2.** No colonization of C. albicans was found in placental tissue in mice infected with C. albicans. Three placental samples in each group at E11.5 were amplified using three pairs the ITS region of rRNA gene primers. No amplification was seen on the gel.

**
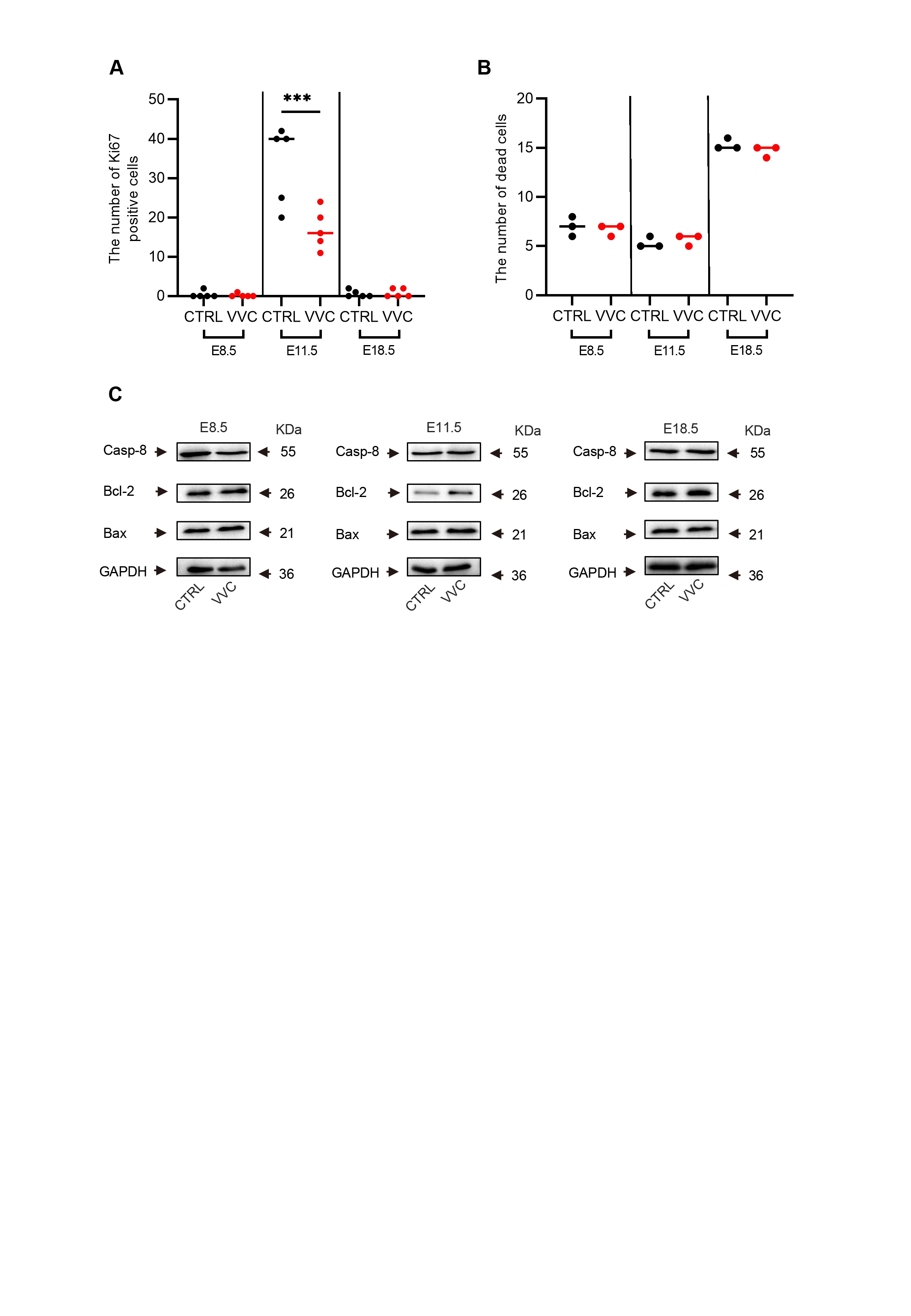
**

**Supplementary Figure 3.** Effect of vaginal C. albicans infection on the proliferation and apoptosis of placenta. (A and B) The number of Ki67 positive cells and dead cells at E8.5, E11.5, E18.5 (n=5). (C) Representative immunoblotting results of Bax, Bcl-2, and Caspase-8 proteins at E8.5 and E11.5 (n=3). Data are shown as mean ± SD. Unpaired t-test was used for two-group comparisons. ****p* < 0.001.
